# *Streptococcus suis* Infection and Malignancy in Man, Spain

**DOI:** 10.3201/eid2006.131167

**Published:** 2014-06

**Authors:** Silvia Gómez-Zorrilla, Carmen Ardanuy, Jaime Lora-Tamayo, Jordi Cámara, Dolors García-Somoza, Carmen Peña, Javier Ariza

**Affiliations:** University of Barcelona, Barcelona, Spain

**Keywords:** Streptococcus suis, cancer, malignancy, immunosuppression, meningitis, arthritis, diplopia, bacteria, zoonoses

**To the Editor:** *Streptococcus suis* is an emerging zoonotic agent. Human infection is associated with occupational exposure to swine. Affected persons are usually, but not always, healthy (*1*,*2*). Immunosuppressive conditions can predispose persons to *S. suis* infection, and cancer has classically been associated as a risk factor for *S. suis* infection (*1*,*2*). Nevertheless, the actual number of reported cases is low (*2*–*7*). We describe a severe case of *S. suis* infection in a man who had not been exposed to swine but for whom disseminated cancer was diagnosed 5 months after the infection.

In 2012, a 57-year-old alcoholic man from Spain, who had no other medical conditions and no contact with animals sought care for headache and vomiting for 24 hours. He reported a 4-day history of fever and a painful right shoulder. At admission, temperature was 38.9°C, blood pressure 180/100 mm Hg, heart rate 68 beats/min, and respiratory rate 24 breaths/min. Neck stiffness and lethargic mental status were noted.

Laboratory tests revealed the following values: leukocytosis of 14 × 10^9^ (reference range 3.9–10 × 10^9^) cells/L with 90.4% neutrophils, platelets 100×10^9^ (reference 135–333 × 10^9^) cells/L, hemoglobin 16 (reference 12.6–16.6) g/dL, creatinine 131 (reference 0–111) μmol/L, and C-reactive protein 243 (reference 0–5) mg/L. Lumbar puncture yielded turbid cerebrospinal fluid (CSF), with high opening pressure (>32 cm H_2_O), pleocytosis (0.4 × 10^9^ leukocytes/L; 88% neutrophils), high protein level (70 [reference range 15–45] mg/dL) and a low glucose level (<0.3 [reference 2.2–4.1] mmol/L). CSF showed gram-positive cocci in chains. Cefotaxime, dexamethasone, and mannitol were administered. After septic shock and respiratory insufficiency developed, the patient was transferred to the intensive care unit.

*Streptococcus* spp. grew in blood and CSF cultures. Although initially misidentified as *S. bovis,* the pathogen was confirmed as *S. suis* by sequence analysis of the 16S rRNA gene. Multilocus sequence typing (http://ssuis.mlst.net) identified this isolate as sequence type (ST) 3. 

The patient was transferred to the medical ward 18 days after admission. Neurologic examination demonstrated vestibular ataxia, hearing loss, and diplopia resulting from cranial nerve VI palsy. Furthermore, a diagnosis of subacromial/subdeltoid bursitis led to arthroscopic debridement. Ceftriaxone was administered for 4 weeks. Results of abdominal computed tomography and echocardiogram were within normal limits. Because the *Streptococcus* organism was initially identified as *S. bovis*, colonoscopy and assessment of tumor markers were also requested; results were within normal limits.

After the patient was discharged (4 weeks after admission), diplopia and the shoulder mobility limitation completely resolved, but bilateral deafness and ataxia persisted. Five months later, the patient was readmitted for severe hypercalcemia. Positron-emission and computed tomography revealed liver, lung, and bone metastases. Tumor markers were elevated (carcinoembryonic antigen 4,152 [reference range 0–4.3] μg/L; monoclonal antibody CA-19–1 9,233 [0–39] U/mL). The patient died of multiorgan failure 21 days after admission. Necropsy revealed a disseminated esophageal adenocarcinoma.

*S. suis* is an encapsulated gram-positive, catalase-negative facultative anaerobe coccus, positive for Lancefield group antigens R-S or T. This pathogen of swine is infrequently transmitted to humans (*1*–*3*,*6*); recently, however, the number of *S. suis* cases in humans has increased substantially. Most cases have been reported in Europe and Southeast Asia, where pig farming is intensive (*1*). Although cases are usually sporadic, 2 outbreaks in China (1998 and 2005) caused a substantial number of deaths. Exposure to infected pigs was demonstrated for almost all patients. However, some patients had not been exposed to animals (*1*,*2*,*4*). *S. suis* can be an opportunistic pathogen in immunocompromised persons (*1*,*2*). Splenectomy is a well-established risk factor. Other predisposing factors are alcoholism, heart disease, and diabetes (*1*,*4*,*5*).

Although cancer is accepted as a risk factor (*1*,*2*), the reported number of cases with associated malignancy is quite low **(**Table)**.** For all cases except one, cancer was diagnosed before or during the episode of infection. A primary adrenal lymphoma was diagnosed 1 year after *S. suis* meningitis (*6*), but probably an underlying defect in humoral immunity was already present. The patient reported here probably had subclinical malignancy at the time of infection. Although we cannot rule out a spurious relationship between cancer and infection, we believe that malignancy, in combination with the patient’s alcoholism, led to an immunosuppressed condition that facilitated the development of infection.

**Table:** Cases of *Streptococcus suis* infection and cancer reported in the literature*

Year	Country of origin	Patient age, y/sex	Clinical presentation	Malignancy	Time of malignancy diagnosis†	Animal contact	Infection outcome	Ref.
2012	Spain	57/M	Meningitis, arthritis, bacteremia	Disseminated esophageal adenocarcinoma	5 mo after *S. suis* infection.	No	Survived	This study
2007	Italy	68/M	Meningitis	Lung squamous cell carcinoma	At admission	No	Survived	(*2*)
2006	Greece	59/M	Endocarditis	Colon carcinoma	At admission	Yes (farmer)	Survived	(*3*)
2004	Hong Kong	81/F	Cellulitis, bacteremia	Breast malignancy	Before *S. suis* infection	Yes	Survived	(*4*)
2001	Thailand	NA	NA	Stomach cancer	Before *S. suis* infection	NA	NA	(*5*)
1994	Taiwan	61/M	Meningitis	Primary adrenal B-cell lymphoma	1 y after *S. suis* infection.	Yes (pig farmer)	Survived	(*6*)
1983	The Netherlands	76/M	Meningitis	Pancreatic carcinoma	Before *S. suis* infection	Yes (pig keeper)	Survived	(*7*)
1981	The Netherlands	52/M	Meningitis	Stomach carcinoma	Before *S. suis* infection	Yes (cut meat for dog)	Survived	(*7*)

*Ref., reference; NA, not available. †With regard to the episode of *S. suis* infection.

*S. suis* leads to a wide spectrum of clinical manifestations, meningitis being the most common (*1*–*3*,*6**–**8*). A higher frequency of sensorineural hearing loss is characteristic of *S. suis* meningitis (*1*). In the patient reported here, meningitis was complicated by permanent deafness, ataxia, and transient diplopia; to our knowledge, only 2 other cases complicated by diplopia have been reported (*8**,**9*).

*S. suis* ST3 belongs to ST clonal complex 1 and is associated with serotype 2 (http://ssuis.mlst.net). Although clonal complex 1 accounts for most *S. suis* infections in humans (*1*,*10*), genotype ST3 is extremely rare. To our knowledge, only 1 other human case of *S. suis* ST3 infection has been reported, also in Spain (*10*).

The patient reported here had severe *S. sui*s infection with no prior exposure to swine but with undiagnosed neoplasia. In patients with no exposure to swine, we recommend searching for other predisposing factors, such as malignancy or other immunodeficiencies.
